# Supplementary material for: Serum Proteomics Reveals Diagnostic Biomarkers and Molecular Pathways in Cerebral Palsy
Source: Nat Commun. 2025 Nov 21;16:10253. doi: 10.1038/s41467-025-65110-6 (PMC12638935; doi:10.1038/s41467-025-65110-6)
Supplement: Supplementary file 1 — Supplementary Information [file 41467_2025_65110_MOESM1_ESM.pdf]

Figure S1

A

Characteristics	CP (n =346)	HC (n = 190)	*P*
Gender			
Female	106 (30.8%)	49 (26%)	0.233
Male	238 (69.2%)	141 (74%)	
Unknown	2		
Age median (month)	23	24	0.16

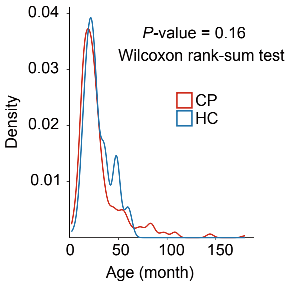

B

Characteristics	CP
Subtype	
Ataxic	9 (2.7%)
Dyskinetic	17 (4.9%)
Mixed	40 (11.6%)
Spastic	278 (80.8%)
Unknown	2
GMFCS	
Level I	90 (26.2%)
Level II	84 (24.4%)
Level III	60 (17.4%)
Level IV	47 (13.7%)
Level V	63 (18.3%)
Unknown	2
MRI	
Maldevelopments	18 (6.1%)
Predominant white matter injury	183 (61.8%)
Predominant gray matter injury	43 (14.5%)
Miscellaneous	29 (9.8%)
Normal	23 (7.8%)
Unknown	50

Risk factors	CP
Adverse pregnancy history	21 (6.1%)
Threatened abortion	45 (13.2%)
Muliple pregnancy	35 (10.2%)
Placental abnormality	15 (4.4%)
Fetal distress	6 (1.7%)
Pregnancy−related complications	58 (16.9%)
Umbilical cord around neck	58 (16.9%)
Abnormal amniotic fluid	45 (13.1%)
Perinatal asphyxia	93 (27.1%)
Low birth weight	101 (30.2%)
Preterm birth	118 (34.6%)
PVL	130 (37.8%)
Pathological jaundice	102 (29.7%)
Intracranial hemorrhage	17 (4.9%)
Neonatal hypoglycemia	27 (7.8%)

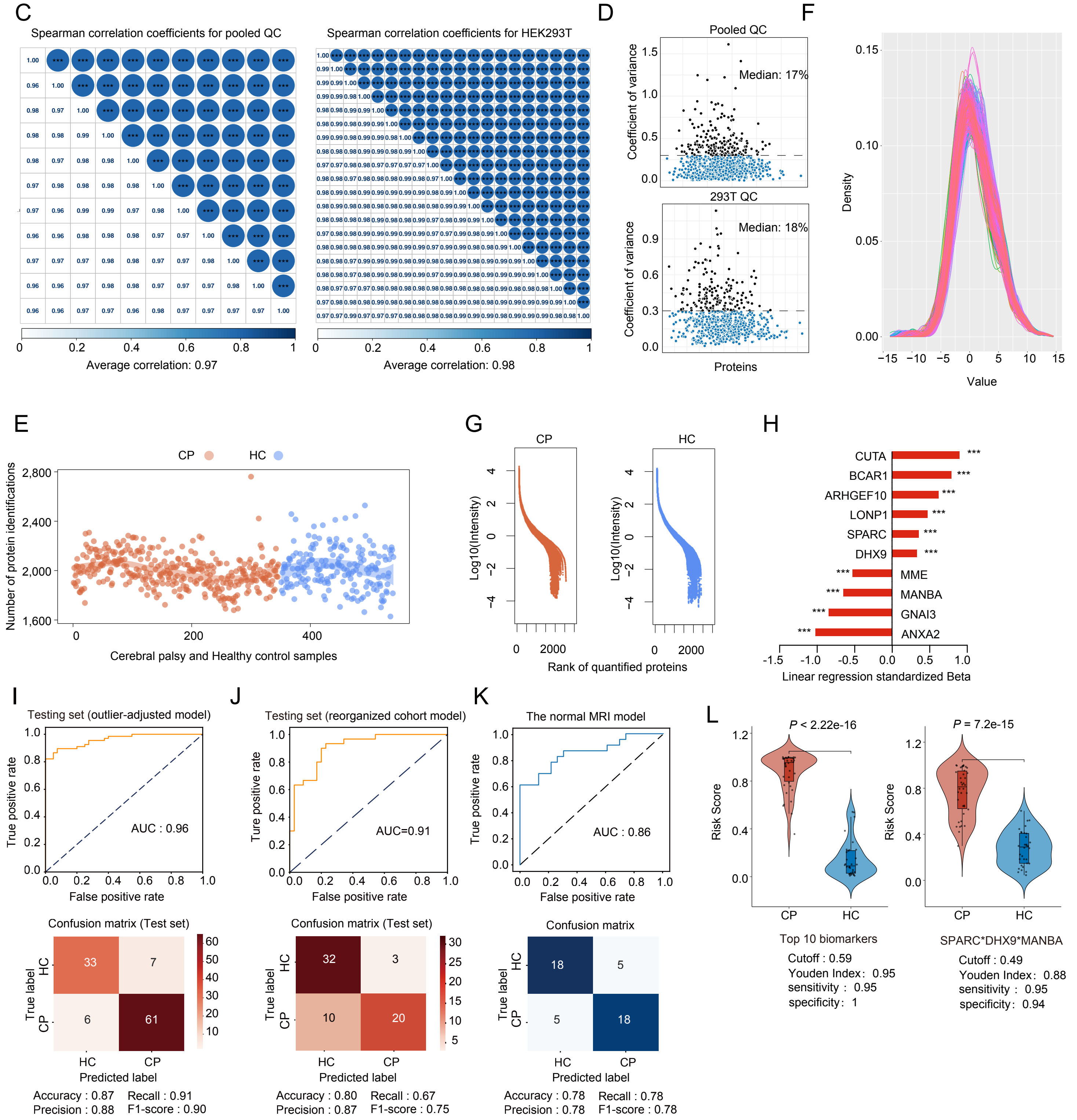

Supplementary Figure Legends

Figure S1. **Overview of the serum proteome profiling of patients with CP. Related to Figure 1**. **A**. Age and sex distribution in the study cohort. Fisher's exact test was used to compare sex distribution between the CP and HC groups, and the Wilcoxon rank-sum test was used to compare age distribution. *P* value was derived by the two-sided test. Sample distribution: between the CP (n=344) and HC groups (n=190). **B**. Tables showing the demographic and clinical information of CP patients. **C**. Spearman's correlation analysis of HEK293T cell QC (n=20) and serum pooled QC (n=11). *P* value was derived by the two-sided test. $*P < 0.05$; $** P < 0.01$; $*** P < 0.001$. **D**. Coefficient of variance of proteins of serum pooled QC and HEK293T QC. The blue dot indicated the CVs less than 30%. **E**. Proteins identified in both CP and healthy control serum samples. The red dots indicate CP individuals, and the blue dots indicate HC. **F**. Distribution of protein abundances across all samples using a density plot (n=536). **G**. Dynamic range of the protein identification of each sample according to the descending sort of protein abundance across all samples. **H**. Bar plot illustrating the regression coefficients of disease (FDR < 0.05). **I**. ROC curves evaluating the performance of the 10 combined biomarkers in outlier-adjusted models, accompanied by confusion matrices illustrating the performance of the machine learning classifier. **J**. Up: ROC curve depicting the AUC for the XGBoost models. Down: Confusion matrix showing the performance of the machine learning classifier on the 20% testing set. K. ROC curve evaluating the performance of the 10 combined biomarkers in MRI-normal CP patients, accompanied by a confusion matrix illustrating the performance of the machine learning classifier. **L.** Left: Boxplot displaying the risk score distribution based on the 10 biomarkers derived from ELISA results. Right: Boxplot showing the risk score based on SPARC*DHX9*MANBA panel. Sample distribution: between the CP (n=38) and HC groups (n=32). Boxplots show median (central line), upper and lower quartiles (box limits), 1.5×interquartile range (whiskers). Source data are provided as a Source Data file.

Figure S2

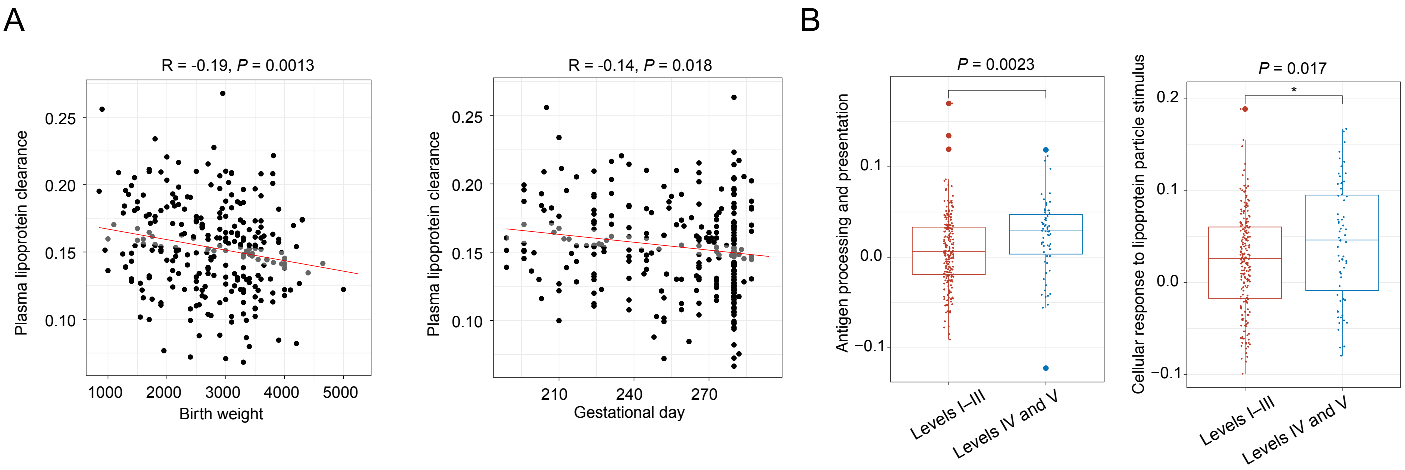
A
R = -0.19, P = 0.0013
Plasma lipoprotein clearance
0.25
0.20
0.15
0.10
1000
2000
3000
4000
5000
Birth weight
R = -0.14, P = 0.018
Plasma lipoprotein clearance
0.25
0.20
0.15
0.10
210
240
270
Gestational day
B
P = 0.0023
Antigen processing and presentation
0.1
0.0
−0.1
Levels I–III
Levels IV and V
P = 0.017
*
Cellular response to lipoprotein particle stimulus
0.2
0.1
0.0
−0.1
Levels I–III
Levels IV and V

Figure S2. **Characteristics of birth weight and gestation day**. **A**. Scatterplot showing the relationship between pathway ssGSEA score and birth weight and gestational day (Spearman's correlation) at protein level. *P* value was derived by the two-sided test. **B**. Boxplots show significant pathway score between high and low GMFCSat protein level (two-sided Wilcoxon rank-sum test). Sample distribution: GMFCS level I- III (n=215) and level IV- V (n=63). Boxplots show median (central line), upper and lower quartiles (box limits), 1.5×interquartile range (whiskers). Source data are provided as a Source Data file.

Figure S3

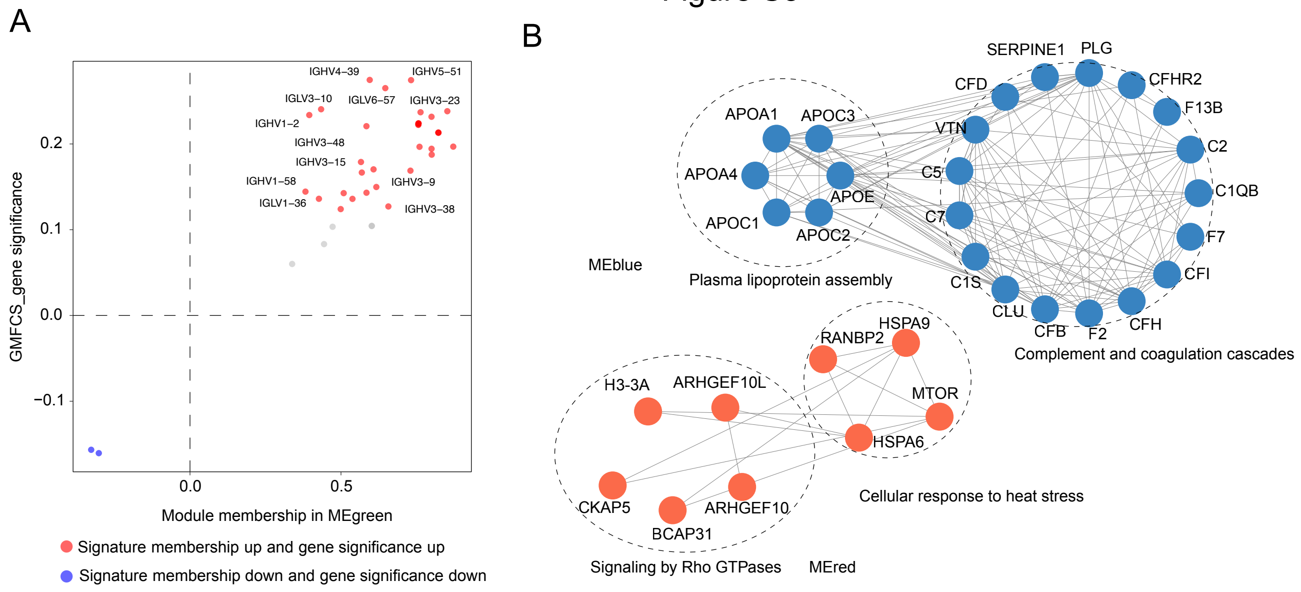

A
B
IGHV4−39
IGHV5−51
IGLV3−10
IGLV6−57
IGHV3−23
IGHV1−2
IGHV3−48
IGHV3−15
IGHV3−9
IGHV1−58
IGLV1−36
IGHV3−38
GMFCS_gene significance
0.2
0.1
0.0
−0.1
0.0
0.5
Module membership in MEgreen
Signature membership up and gene significance up
Signature membership down and gene significance down
SERPINE1
PLG
CFD
CFHR2
F13B
APOA1
APOC3
VTN
C2
APOA4
C5
APOE
C1QB
C7
APOC1
APOC2
F7
MEblue
Plasma lipoprotein assembly
C1S
CFI
CLU
CFB
F2
CFH
Complement and coagulation cascades
RANBP2
HSPA9
H3-3A
ARHGEF10L
MTOR
HSPA6
CKAP5
ARHGEF10
BCAP31
Cellular response to heat stress
Signaling by Rho GTPases
MEred

Figure S3. **Protein co-expression classifies showing the characteristic proteomic profiles. Related to Figure 5**. **A**. Scatterplot showing the correlation between proteins in the MEgreen module and GMFCS. The module membership represents the correlation of each protein with its respective module (x-axis), and the gene significance (GS) represents the correlation between proteins and clinical traits (y-axis). **B**. The protein-protein interaction networks constructed by the proteins enriched in specific modules. The red dots indicate proteins in MEred, and the blue dots indicate proteins in MEblue. Source data are provided as a Source Data file.
