# Supplementary material for: Serum Proteomics Reveals Diagnostic Biomarkers and Molecular Pathways in Cerebral Palsy
Source: Nat Commun. 2025 Nov 21;16:10253. doi: 10.1038/s41467-025-65110-6 (PMC12638935; doi:10.1038/s41467-025-65110-6)
Supplement: Supplementary file 2 — Description of Additional Supplementary Files [file 41467_2025_65110_MOESM2_ESM.pdf]

Description of Additional Supplementary Files

File Name: Supplementary Data 1
Description:

1. Clinical traits: Baseline characteristics of participants included in the study. The three-line table within the file details the clinical characteristics of the cohorts, with continuous variables shown as mean and categorical variables as number (percentage).

2. Protein expression: The protein expression matrix of healthy control (HC) (n=190) and CP (n=346) serum samples.

3. DEP: The differentially expressed proteins between CP and HC.

4. The P/LP variants: Loci of pathogenic/likely pathogenic (P/LP) variants.

5. WGCNA: Each protein was assigned to a specific module, and its module membership (MM) value was calculated as the correlation between the protein expression profile and the module eigengene. The table summarizes module assignments for all proteins, including MM values across different modules and their corresponding p-values.
