# Supplementary material for: Serum Proteomics Reveals Diagnostic Biomarkers and Molecular Pathways in Cerebral Palsy
Source: Nat Commun. 2025 Nov 21;16:10253. doi: 10.1038/s41467-025-65110-6 (PMC12638935; doi:10.1038/s41467-025-65110-6)
Supplement: Supplementary file 4 — Reporting Summary [file 41467_2025_65110_MOESM4_ESM.pdf]

nature portfolio

Corresponding author(s): Chen Ding, Changlian Zhu

Last updated by author(s): Sep, 9, 2025

Reporting Summary

Nature Portfolio wishes to improve the reproducibility of the work that we publish. This form provides structure for consistency and transparency in reporting. For further information on Nature Portfolio policies, see our Editorial Policies and the Editorial Policy Checklist.

Statistics

For all statistical analyses, confirm that the following items are present in the figure legend, table legend, main text, or Methods section.

n/a	Confirmed	
☐	☒	The exact sample size (*n*) for each experimental group/condition, given as a discrete number and unit of measurement
☐	☒	A statement on whether measurements were taken from distinct samples or whether the same sample was measured repeatedly
☐	☒	The statistical test(s) used AND whether they are one- or two-sided<br>*Only common tests should be described solely by name; describe more complex techniques in the Methods section.*
☐	☒	A description of all covariates tested
☐	☒	A description of any assumptions or corrections, such as tests of normality and adjustment for multiple comparisons
☐	☒	A full description of the statistical parameters including central tendency (e.g. means) or other basic estimates (e.g. regression coefficient) AND variation (e.g. standard deviation) or associated estimates of uncertainty (e.g. confidence intervals)
☐	☒	For null hypothesis testing, the test statistic (e.g. *F*, *t*, *r*) with confidence intervals, effect sizes, degrees of freedom and *P* value noted<br>*Give P values as exact values whenever suitable.*
☒	☐	For Bayesian analysis, information on the choice of priors and Markov chain Monte Carlo settings
☐	☒	For hierarchical and complex designs, identification of the appropriate level for tests and full reporting of outcomes
☐	☒	Estimates of effect sizes (e.g. Cohen's *d*, Pearson's *r*), indicating how they were calculated

Our web collection on statistics for biologists contains articles on many of the points above.

Software and code

Policy information about availability of computer code

Data collection	The serum proteomic raw data were performed by by Q Exactive HF-X Mass Spectrometer, Thermo Fisher Scientific.
Data analysis	All mass spectrometry raw files from serum (DIA mode) samples were processed through the Firmiana cloud platform. The DIA data analysis was conducted through two complementary approaches: (1) database searching with FragPipe (v12.1) utilizing MSFragger (v2.2), and (2) spectral library-based analysis with DIA-NN (v1.7.0), both implemented within the Firmiana environment.<br><br>The data analysis was performed in in the R (v4.1.2) and python (v3.1.0) environments with open-source libraries. R Packages: GSVA (version 1.42.0), WGCNA (1.72.1), limma (3.50.3), impute (1.68.0). Python Packages: XGBoost (version 2.1.0). Pathway enrichment analysis was conducted with online tools ConsensusPathDB (http://cpdb.molgen.mpg.de/).

For manuscripts utilizing custom algorithms or software that are central to the research but not yet described in published literature, software must be made available to editors and reviewers. We strongly encourage code deposition in a community repository (e.g. GitHub). See the Nature Portfolio guidelines for submitting code & software for further information.

Data

Policy information about availability of data

All manuscripts must include a data availability statement. This statement should provide the following information, where applicable:

- Accession codes, unique identifiers, or web links for publicly available datasets
- A description of any restrictions on data availability
- For clinical datasets or third party data, please ensure that the statement adheres to our policy

The raw mass spectrometry proteomic data generated in this study have been deposited to the ProteomeXchange Consortium (https://proteomecentral.proteomexchange.org) via the iProX partner repository, with subproject IDs PXD068404. Additional processed data supporting the findings are available in the Supplementary Information or Source Data files. Source data are provided with this paper.

Research involving human participants, their data, or biological material

Policy information about studies with human participants or human data. See also policy information about sex, gender (identity/presentation), and sexual orientation and race, ethnicity and racism.

Reporting on sex and gender	This study does not pertain to issues of sex and gender.
Reporting on race, ethnicity, or other socially relevant groupings	The participants in our study are all Chinese people and they were not classified into subgroups based on their race, ethnicity, or other socially relevant grouping.
Population characteristics	All patients were classified into clinical subtypes of CP according to ICD-10 codes, including dyskinetic subtype (n=17), spastic subtype (n=278), ataxic subtype (n=9), mixed subtype (n=40). The median ages of the CP and healthy controls (HC) were 23 and 24 months. The sex distribution did not significantly differ between groups (CP: 69.2% male; HC: 74% male).
Recruitment	The clinical cohorts of Chinese children diagnosed with CP from two medical centers: the Children's Hospital and the Third Affiliated Hospital of Zhengzhou University. All children diagnosed with CP met Surveillance of Cerebral Palsy in Europe (SCPE) criteria. The inclusion criteria: 1) All patients were under 18 years of age. For those who received an initial diagnosis before the age of 2, longitudinal follow-up was performed until they reached at least 2 years of age to confirm the stability and accuracy of the diagnosis. 2) Diagnosed with CP following comprehensive clinical evaluations conducted by pediatric neurologists, including pregnancy and birth history, family history, and detailed physical and neurological examinations; 3) No known chromosomal abnormalities or syndromic diagnoses. The exclusion criteria: 1) Unstable vital signs or serious systemic illness; 2) CP secondary to acquired causes (e.g., encephalitis, uncontrolled epilepsy); 3) CP subtypes predominantly characterized by hypotonia or rigidity. The diagnostic classification and clinical information used in this study were determined after the age of 2 years for these early-diagnosed cases.<br><br>Healthy control samples were obtained during routine pediatric medical examinations at the Third Affiliated Hospital of Zhengzhou University. The inclusion criteria: 1) Typically developing children with no history of neurological, genetic, or developmental disorders. 2) No recent perinatal complications or current medications. The exclusion criteria: 1) Recent infections, fever, or vaccination within two weeks; 2) First-degree family history of neurodevelopmental or inherited genetic disorders.
Ethics oversight	Informed consent was obtained from all participants and their guardians, with a thorough understanding of the study's purpose and consent for the publication of the results. The research received approval from the Ethics Committee of The Third Affiliated Hospital of Zhengzhou University (Ethical Approval No. 2017-09) and complied with relevant guidelines and regulations. Healthy controls were recruited from children experiencing kindergarten, ensuring a representative sample of the general population.

Note that full information on the approval of the study protocol must also be provided in the manuscript.

Field-specific reporting

Please select the one below that is the best fit for your research. If you are not sure, read the appropriate sections before making your selection.

☒ Life sciences ☐ Behavioural & social sciences ☐ Ecological, evolutionary & environmental sciences

For a reference copy of the document with all sections, see nature.com/documents/nr-reporting-summary-flat.pdf

Life sciences study design

All studies must disclose on these points even when the disclosure is negative.

Sample size	In this study, high-resolution MS-based serum proteomic analyses was performed on a total of 346 CP patients and 190 healthy controls.
Data exclusions	The exclusion criteria: 1) Unstable vital signs or serious systemic illness; 2) CP secondary to acquired causes (e.g., encephalitis, uncontrolled

Data exclusions	epilepsy); 3) CP subtypes predominantly characterized by hypotonia or rigidity.
Replication	Strict quality control protocols were implemented to ensure the reliability of MS performance and the entire experimental process. Specifically, pooled serum samples, mixed by all CP and HC samples in our study, were included to monitor overall technical consistency, while human embryonic kidney-derived HEK293T cell lysates served as external QC standards to evaluate platform stability and performance across runs. The preparation of pooled QC samples followed the same protocol as the cohort serum samples. This resulted in a total of 20 HEK293T QC runs and 11 pooled QC runs. Mass spectrometry assays for both the pooled QC and HEK293T samples were aligned with those of the disease and control samples to ensure consistency.<br><br>An independent cohort (38 CP children and 32 healthy controls) were collected to validate the cross-platform reproducibility of the candidate biomarkers.
Randomization	In this study, randomization was applied to both the collection of clinical samples and the acquisition of proteomic data. Clinical samples were randomized after following predefined inclusion and exclusion criteria, ensuring unbiased selection. The proteomic data acquisition was also fully randomized, minimizing potential biases in the outcomes.
Blinding	In this study, rigorous blinding procedures were implemented throughout the experimental workflow. Clinical samples were collected by unblinded clinical staff according to predefined inclusion and exclusion criteria. However, laboratory investigators measuring protein expression were blinded to patient clinical information during data collection and analysis. Similarly, all bioinformatics analyses were conducted under blinded conditions.

Reporting for specific materials, systems and methods

We require information from authors about some types of materials, experimental systems and methods used in many studies. Here, indicate whether each material, system or method listed is relevant to your study. If you are not sure if a list item applies to your research, read the appropriate section before selecting a response.

Materials & experimental systems

n/a	Involved in the study	
☒	☐	Antibodies
☐	☒	Eukaryotic cell lines
☒	☐	Palaeontology and archaeology
☒	☐	Animals and other organisms
☒	☐	Clinical data
☒	☐	Dual use research of concern
☒	☐	Plants

Methods

n/a	Involved in the study	
☒	☐	ChIP-seq
☒	☐	Flow cytometry
☒	☐	MRI-based neuroimaging

Eukaryotic cell lines

Policy information about cell lines and Sex and Gender in Research

Cell line source(s)	The HEK293T cell line (ATCC CRL-11268, RRID: CVCL_QW54) was obtained from the Chinese Academy of Sciences.
Authentication	All cell lines were routinely tested for mycoplasma contamination and authenticated by Short Tandem repeat (STR) profiling.
Mycoplasma contamination	All cell lines tested negative for mycoplasma contamination.
Commonly misidentified lines (See ICLAC register)	No commonly misidentified cell lines were used.

Plants

Seed stocks	No No plants were used.
Novel plant genotypes	No No plants were used.
Authentication	No No plants were used.
